# Hydrogen Improves Glycemic Control in Type1 Diabetic Animal Model by Promoting Glucose Uptake into Skeletal Muscle

**DOI:** 10.1371/journal.pone.0053913

**Published:** 2013-01-10

**Authors:** Haruka Amitani, Akihiro Asakawa, Kaichun Cheng, Marie Amitani, Kaori Kaimoto, Masako Nakano, Miharu Ushikai, Yingxiao Li, Minglun Tsai, Jiang-Bo Li, Mutsumi Terashi, Huhe Chaolu, Ryozo Kamimura, Akio Inui

**Affiliations:** 1 Department of Psychosomatic Internal Medicine, Kagoshima University Graduate School of Medical and Dental Sciences, Kagoshima, Japan; 2 Institute of Laboratory Animals, Kagoshima University Graduate School of Medical and Dental Sciences, Kagoshima, Japan; Virgen Macarena University Hospital, School of Medicine, Spain

## Abstract

Hydrogen (H_2_) acts as a therapeutic antioxidant. However, there are few reports on H_2_ function in other capacities in diabetes mellitus (DM). Therefore, in this study, we investigated the role of H_2_ in glucose transport by studying cultured mouse C2C12 cells and human hepatoma Hep-G2 cells *in vitro*, in addition to three types of diabetic mice [Streptozotocin (STZ)-induced type 1 diabetic mice, high-fat diet-induced type 2 diabetic mice, and genetically diabetic *db/db* mice] *in vivo*. The results show that H_2_ promoted 2-[^14^C]-deoxy-d-glucose (2-DG) uptake into C2C12 cells via the translocation of glucose transporter Glut4 through activation of phosphatidylinositol-3-OH kinase (PI3K), protein kinase C (PKC), and AMP-activated protein kinase (AMPK), although it did not stimulate the translocation of Glut2 in Hep G2 cells. H_2_ significantly increased skeletal muscle membrane Glut4 expression and markedly improved glycemic control in STZ-induced type 1 diabetic mice after chronic intraperitoneal (i.p.) and oral (p.o.) administration. However, long-term p.o. administration of H_2_ had least effect on the obese and non-insulin-dependent type 2 diabetes mouse models. Our study demonstrates that H_2_ exerts metabolic effects similar to those of insulin and may be a novel therapeutic alternative to insulin in type 1 diabetes mellitus that can be administered orally.

## Introduction

Diabetes mellitus (DM) is characterized by abnormal insulin secretion, derangements in carbohydrate-lipid metabolism, and chronic hyperglycemia. The total number of people with DM is predicted to rise from 171 million in 2000 to 366 million in 2030 [1]. Therefore, various therapies, including alternative medicine, are being developed for DM. Insulin-like growth factor 1 (IGF-1) exerts metabolic effects similar to those of insulin [2], [3]. Although IGF-1 was once thought to have the same effect as insulin, recently a relationship between IGF-1 and diabetic retinopathy has been suggested [4]. Indeed, the only treatment for progressed DM is insulin therapy.

Insulin initiates its action by binding its specific cell-surface receptor, insulin receptor (IR), in the peripheral tissues such as skeletal muscle and liver under normal conditions. IR is a heterotetrameric protein that consists of two extracellular α-subunits and two transmembrane β-subunits connected by disulfide bridges. Insulin signaling is initiated following binding of insulin to the extracellular α-subunits. This results in autophosphorylation of the β-subunits and activation of the tyrosine kinase domain of the intracellular part of the β subunits [5]. The active tyrosine kinase of IR phosphorylates IR substrate (IRS) proteins. Then, the phosphorylated IRS proteins recruit and activate phosphatidylinosisitol 3-kinase (PI3K). IR, IRS, and PI3K are considered critical nodes in insulin signaling [6]. The activated PI3K activates its downstream effectors, such as Akt and protein kinase C (PKC), by increasing the production of its lipid product, phosphatidylinositol-3,4,5-(PO_4_)_3_, which leads to glucose uptake.

Hydrogen (H_2_) is the lightest gas molecule. The numerous strains of intestinal bacteria, primarily in the large intestine, produce H_2_, and approximately 14% of H_2_ is absorbed in the colon and released from the lungs [7]. The hydrogen breath test has become popular in clinical practice because it is useful to assess abnormal pathophysiology, such as bacterial overgrowth in the small intestine, and to diagnose lactose or fructose malabsorption [8]. Gharib et al reported the antioxidant effect of H_2_ using mice with parasite-induced liver inflammation [9]. Ohsawa et al recently reported that hydrogen acts as a therapeutic anti-oxidant by selectively reducing hydroxyl radicals (·OH) [10]. H_2_ affects ischemia-reperfusion injury [10], [11], atherosclerosis [12], Parkinson’s disease [13], acute pancreatitis [14], and type 1 allergic reaction [15]. Currently, there are more than 100 reports related to antioxidant effects of H_2_.

Although several studies have described the antioxidant effect of H_2_ on DM [16], [17], few studies have reported on other functions of H_2_ in DM. Therefore, in this study, we investigated the role of H_2_ in glucose homeostasis by studying cultured mouse C2C12 cells and human hepatoma Hep G2 cells *in vitro*, as well as three types of diabetic mice (STZ-induced diabetic mice, high-fat diet induced diabetic mice, and genetically diabetic *db/db* mice) *in vivo*.

## Materials and Methods

### Cell Culture

The C2C12 cells and Hep G2 cells were obtained from Bioresource Collection and Research Center (Food Industry Research and Development Institute, Hsinchu City, Taiwan). The cells were plated at 5×10^4^ cells/dish in 35-mm-diameter culture dishes in Dulbecco’s modified Eagle’s medium (DMEM) (Gibco BRL, Gaithersburg, USA) supplemented with 10% fetal bovine serum (FBS) (Gibco BRL) and 1% antibiotic solution (penicillin G sodium 10,000 U/ml and streptomycin sulfate 10 µg/ml). They were grown to 70% confluence at 37°C in humidified atmosphere containing 5% CO_2_. To induce fusion, confluent cells were exposed to DMEM supplemented with 10% horse serum instead of FBS. The cells fused into multinucleated myotubes after a further 7–10 days in culture. The medium was changed 24 h prior to experimental manipulations.

### H_2_-containing Water and Saline

High-content (saturated) H_2_ water and saline (HHW and HHS, respectively: 0.8 mM each) were prepared by dissolving H_2_ in pure water or saline under high pressure (0.4 MPa) for 24 hours. Low-content H_2_ water (LHW: 0.08 mM) was prepared by dissolving H_2_ in pure water under low pressure (0.1 MPa) for 24 hours. Artificial H_2_ water and saline were made every day. In this study, we also used natural H_2_ water (NHW) drawn from Mt. Fuji (Yamanashi, Japan). NHW containing 0.075–0.125 mM H_2_ was provided by VANA Co., Ltd. (Yamanashi, Japan) every day. LHW, HHW and NHW were placed in a glass vessel as drinking water for oral administration (p.o.). HHW and NHW were also used *in* *vitro* experiments. On the other hand, HHS was used for intraperitoneal (i.p.) injection. H_2_ content was measured with a hydrogen electrode (DH-35A, TOA DKK Co. Ltd., Tokyo, Japan).

### H_2_ Attenuation in Pure Water and Several Solutions

We speculated that NHW might keep its H_2_ content longer than LHW. NHW contains several solutes such as sodium (0.2 mM), calcium (0.19 mM), magnesium (0.16 mM), potassium (0.02 mM), and silicon (0.53 mM), making an almost 1.1 mM solution. To assess H_2_ attenuation, we measured the H_2_ content of LHW, HHW, NHW, and 0.17 mM (0.001%) and 0.34 mM (0.002%) saline water containing 0.8 mM H_2_ after 0, 12 and 24 hours incubation in a glass drinking vessel.

### 2, 2-diphenyl-1-picryl-hydrazyl-hydrate (DPPH) Radical Scavenging Photometric Assay

Free radical scavenging activities of H_2_, NAC and vitamin C were measured by the DPPH assay. Twenty µl of each sample solution [10 mM of NAC (Sigma), 10 mM of vitamin C (Sigma), 0.8 mM of H_2_] and 200 µl of DPPH solution [2 mM of 2, 2-diphenyl-1-picryl-hydrazyl (Sigma) prepared in methanol] were added to each micro plate. Incubated in dark at room temperature for 5 min, the absorbance (Ab) was measured at 540 nm using microplate reader. Radical scavenging activity was calculated as follows: % antioxidant activity = [1−(Ab of sample/Ab of blank)]×100.

### Glucose Uptake

Glucose uptake was determined by measuring the uptake of 2-[^14^C]-deoxy-d-glucose (2-DG) (323 mCi/mmol) (New England Nuclear, Boston, MA, USA) into C2C12 cells, as described previously [18]. In brief, the cells were washed with phosphate-buffered saline (PBS) containing 135 mmol/l NaCl, 2.7 mmol/l KCl, 8 mmol/l Na_2_HPO_4_, 1.4 mmol/l KH_2_PO_4_, 0.5 mmol/l MgC_l2_, 0.7 mmol/l CaCl_2_ and 22 mmol/l glucose. After incubation in serum-free and high-glucose (25 mmol/l) DMEM for 5 h, the cells were transferred to fresh incubation flasks with or without pharmacological inhibitors at the indicated concentrations for 30 min at 37°C. LY-2940002, a PI3K inhibitor (Sigma, St. Louis, MO, USA); chelerythrine, a PKC inhibitor (Sigma); and Compound C (6-[4-(2-piperidin-1-ylethoxy)-phenyl]-3-pyridin-4-ylpyrazolo[1,5-a] pyrimidine), an AMPK inhibitor (Sigma), were used as pharmacological inhibitors. The cells were then incubated with 100 µl of pure water, HHW, degassed NHW or NHW at 37°C for another 30 or 60 min under continuous shaking at 40 cycles/min. Degassed NHW was prepared by shaking its container. Then, the cells were further incubated with 2-DG (1 µCi/ml) for 5 min at 37°C. Nonspecific uptake was obtained by parallel determinations in the presence of 20 µmol/l cytochalasin B (Sigma). Uptake was terminated by the addition of ice-cold PBS. After centrifugation, cells were washed twice with ice-cold PBS.

### Animals

Male C57BL/6 mice and genetically diabetic male *db/db* mice (BKS. Cg-+*Leprdb*/+*Leprdb*/Jcl) were purchased at 6 weeks of age from CLEA Japan, Inc. (Tokyo, Japan). All mice were housed individually in an air-conditioned room at 22±2°C with a 12-h light/dark cycle starting at 7∶00 a.m. daily. Mice were used after 1-week acclimatization period. All experimental procedures were performed according to the “Guidelines for the Care and Use of Laboratory Animals” approved by the Kagoshima University Committee for Animal Experiment.

### Chronic i.p. Administration of H_2_ Experiment with STZ Induced Diabetic Mice

Thirty-two male C57BL/6 mice were submitted to i.p. injection of streptozotocin (STZ) (50 mg/kg/day) (Sigma-Aldrich, St. Louis, MO, USA) dissolved in 10 mM Na-citrated buffer (pH4.5) for 5 days to induce diabetes. Control mice (n = 16) were injected with 250 µl of saline and the HHS mice (n = 16) were injected with 250 µl of HHS twice par day for 4 weeks from the first day of i.p. injection of STZ. Body weights and food intake were recorded every day. Plasma glucose was measured every 7 days using a blood glucose meter (NIPRO, Osaka, Japan) with a blood sample obtained by tail prick. On day 32, the mice were sacrificed. The soleus muscle was collected and stored at −80°C until use in the western blot analysis of Glut4.

### Chronic p.o. Administration of H_2_ Experiment with STZ-induced Type 1 Diabetic (T1DM) Mice

Male C57BL/6 mice were submitted to i.p. injection of STZ (50 mg/kg/day) dissolved in 10 mM Na-citrated buffer (pH 4.5) for 5 days. Hyperglycemia was confirmed at 4 weeks post-injection. Only STZ mice with blood glucose above 200 mg/dl were included in the type 1 diabetes mellitus (T1DM) group. A total of 24 mice were divided into four groups: Control (STZ+pure water) (n = 6), STZ+HHW (n = 6), STZ+LHW (n = 6), and STZ+NHW (n = 6). Pure water or hydrogen water was provided for 18 weeks. Body weights and food intake were recorded every day. Plasma glucose was measured every 7 days.

### Chronic p.o. Administration of H_2_ Experiment with High-fat Diet Induced Type 2 Diabetic (T2DM) Mice

The C57BL/6 mouse strain has been used as a model for studies of high-fat diet induced obesity and diabetes [19]. A total of 21 male C57BL/6 mice were fed a high-fat diet (D12492∶60%kcal fat) (Research Diets, Inc., New Brunswick, USA) to induce type 2 diabetes mellitus (T2DM). Seven mice were supplied pure water, HHW or NHW, respectively, for 25 weeks. Plasma glucose was measured every 7 days.

### Chronic p.o. Administration of H_2_ Experiment with Type 2 Diabetic *db/db* Mice

Leptin inhibits food intake by acting on the hypothalamus. This peptide hormone is secreted into the bloodstream from adipose tissues [20] and exerts its effects through the leptin receptor (Ob-R). The Ob-R gene is mutated in *db/db* mice [21], [22], making them unresponsive to leptin, so they exhibit excessively food intake and body weight gain. Therefore, *db/db* mice have been extensively studied as a model of obesity and T2DM. Seven *db/db* mice were supplied pure water, HHW or NHW, respectively, for 18 weeks. Plasma glucose was measured every 7 days.

### Intraperitoneal Glucose Tolerance Test

An intraperitoneal glucose tolerance test (IPGTT) was performed after a 6 h fast. Plasma glucose was measured in tail vein blood at 5, 15, 30, 60 and 120 min after an i.p. injection of 1 mg/g body glucose. IPGTTs were conducted every 30 days.

### Measurements of Biochemical Parameters

All measurements were performed after 6 h of fasting. Blood samples were obtained from the fossa orbitalis venous plexus under diethyl ether anesthesia, transferred to chilled tubes containing ethylenediaminetetraacetic acid, disodium salt (EDTA 2Na) (1 mg/mL) and aprotinin (500 U/mL), and immediately centrifuged. All plasma samples were stored at −80°C until assayed. Glycated albumin was measured with an enzymatic reaction kit (Lucica GA-L, Asahi Kasei Phama Co., Tokyo, Japan). Plasma insulin was measured by an ELISA Insulin kit (Morinaga Co., Tokyo, Japan). Other biochemical parameters were assayed by routine laboratory methods.

### Western Blot Analysis

Total protein lysates from cells or tissues were extracted in lysis buffer (1% Triton X-100, 150 mM NaCl, 10 mM Tris pH 7.5, 5 mM ethylenediaminetetraacetic acid) containing a protease and phosphatase inhibitor cocktail (Sigma-Aldrich). The total protein concentration was determined using a BCA assay kit (Pierce Biotechnology, Rockford, IL, USA). Protein lysates (50 µg) were separated using 10% SDS-polyacrylamide gel electrophoresis and transferred to a polyvinylidene difluoride membrane (Millipore, Billerica, MA, USA). The membrane was blocked at 25°C for 1 h in TBS-T (10 mM Tris pH 7.6, 150 mM NaCl, and 0.05% Tween 20) containing 3% BSA and probed with 1∶1000-diluted primary antibodies against the glucose transporter Glut4 (R&D Systems, Inc., Minneapolis, MN, USA), Glut2 (Abcam Co, Tokyo, Japan), phospho-AMPK (Thr 172) (Cell Signaling Technology, Inc., Beverly, MA, USA), AMPK (Cell Signaling Technology), and actin (Millipore, Billerica, MA, USA) at 4°C overnight. After the membrane had been washed with TBS-T, the blots were incubated with a 1∶5000 dilution of horseradish peroxidase-conjugated secondary antibody at 25°C for 1 h. The protein bands were visualized using an enhanced chemiluminescence kit (PerkinElmer, Boston, MA, USA). Actin was the internal control. The optical densities of the bands were determined using Gel-Pro Analyzer 4.0 software (Media Cybernetics Inc., Silver Spring, MD, USA).

### Real-time RT-PCR

RNA was isolated from the hypothalamic block using the RNeasy Lipid Tissue Mini Kit (Quiagen, K.K., Tokyo, Japan) and the stomach using the RNeasy Fibrous Tissue Midi Kit (Quiagen). Quantification of mRNA was performed with SYBR Green Master (Roche Inc., Basel, Switzerland) using a one-step RT-PCR reaction on a Takara TP800 (Takara Bio Inc., Otsu, Japan). The reaction was performed under standard conditions recommended by the manufacturer. We used the mouse *GAPDH* gene as an internal control. The cycle threshold number (Ct) at which amplification entered the exponential phase was determined for each gene under investigation. Gene expression levels were analyzed using the delta-delta Ct method, determining the target gene expression relative to an internal control and relative to control individual samples. The following primers were used in real-time RT-PCR: GAPDH forward, TCACTGGCATGGCCTTCC; GAPDH reverse, GGCGGCACGTCAGATCC; neuropeptide Y (NPY) forward, TTTCCAAGTTTCCACCCTCATC; NPY reverse, AGTGGTGGCATGCATTGGT; Agouti-related protein (AgRP) forward, GAGTTCCCAGGTCTAAGTCTGAATG; AgRP reverse, ATCTAGCACCTCCGCCAAAG; melanin-concentrating hormone (MCH) forward, GGAAGATACTGCAGAAAGATCCG, MCH reverse, ATGAAACCGCTCTCGTCGTT; orexin forward, CGTAACTACCACCGCTTTAGCA, orexin reverse, TGCCATTTACCAAGAGACTGACAG; pro-opiomelanocortin (POMC) forward, GGCTTGCAAACTCGACCTCT; POMC reverse, TGACCCATGACGTACTTCCG; cocaine- and amphetamine-regulated transcript (CART) forward, GCAGATCGAAGCGTTGCAA; CART reverse, TTGGCCGTACTTCTTCTCGTAGA; corticotropin-releasing factor (CRF) forward, CGCAGCCCTTGAATTTCTTG; CRF reverse, TCTGTTGAGATTCCCCAGGC; Ghrelin forward, TCCAAGAAGCCACCAGCTAA; Ghrelin reverse, AACATCGAAGGGAGCATTGA.

### Statistical Analysis

The results are expressed as the mean values ± standard error (S.E.). Comparisons with controls were performed by unpaired Student’s t test between two groups and Dunnett’s multiple comparison test among more than two groups. P values less than 5% were considered statistically significant.

## Results

### H_2_ Promotes 2-DG Uptake into C2C12 Cells

**Figure 1:**
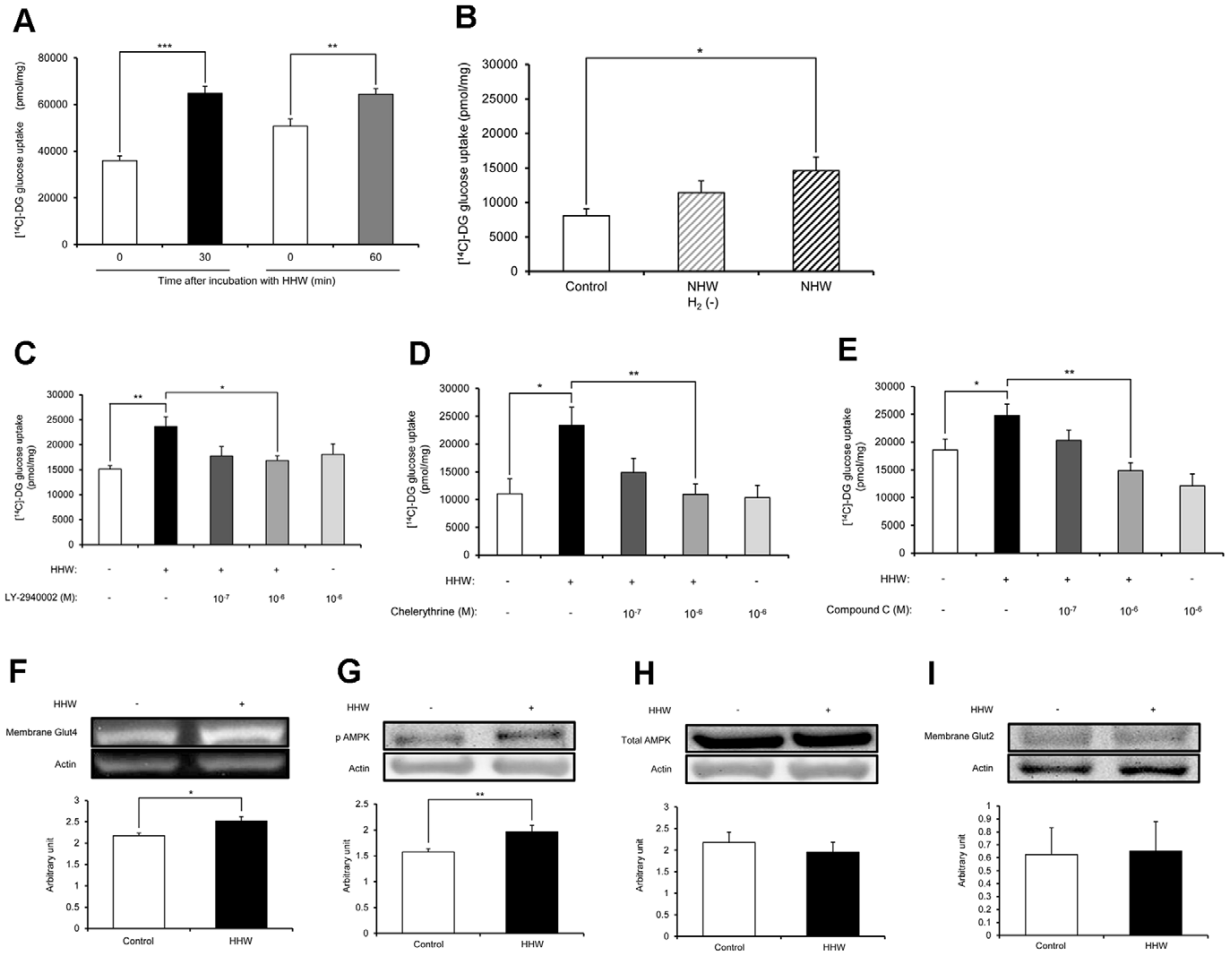
The effect of H_2_ on glucose uptake into C2C12 cells. (A) 2-DG uptake into C2C12 cells after 30 or 60 min exposure to high content hydrogen water (HHW) was significantly increased over control (n = 6 for each group). (B) Natural hydrogen water (NHW) significantly increased 2-DG uptake into C2C12 cells over control, while degassed NHW did not increase 2-DG uptake (n = 7 for each group). (C, D, E) After incubation with or without each pharmacological inhibitor for 30 min, the cells were exposed to pure water or SHW for another 30 min. The addition of LY-2940002, a phosphatidylinositol-3-OH kinase (PI3K) inhibitor, at 1.0 × 10^−6^ M significantly decreased the 2-DG uptake into C2C12 cells compared with HHW alone (n = 6 for each group). The addition of chelerythrine, a protein kinase C (PKC) inhibitor, at 1.0 × 10^−6^ M significantly decreased the 2-DG uptake into C2C12 cells compared with HHW alone (n = 6 for each group). The addition of Compound C (6-[4-(2-piperidin-1-ylethoxy)-phenyl]-3-pyridin-4-ylpyrazolo[1,5-a] pyrimidine), an AMP-activated protein kinase (AMPK) inhibitor, at 1.0 × 10^−6^ M significantly decreased the 2-DG uptake into C2C12 cells compared with HHW alone (n = 10 for each group). (F, G) Western blot analysis was performed as described in the Materials and Methods. HHW increased membrane Glut4 (n = 6 for each group) and phosphorylated AMPK (p-AMPK) (n = 13 for each group) in C2C12 cells. (H, I) There was no significant difference in total AMPK in C2C12 cells (n = 8 for each group) or membrane Glut2 in Hep-G2 cells between groups (n = 6 for each group). Comparisons with controls were performed by unpaired Student’s t test between two groups and Dunnett’s multipule comparison test among more than two groups. *P<0.05, **P<0.01, ***P<0.001.

Figure 1A shows the effect of hydrogen on 2-DG uptake into C2C12 cells. Stimulation of 2-DG uptake into C2C12 cells after 30 and 60 min exposure to HHW was significantly increased over control. NHW also significantly increased the 2-DG uptake into C2C12 cells compared to the control, but degassed NHW did not increase 2-DG uptake (Fig. 1B). The addition of LY-2940002 at 1.0 × 10^−6^ M significantly decreased the 2-DG uptake into C2C12 cells compared with HHW alone (Fig. 1C). The addition of chelerythrine at 1.0 × 10^−6^ M (Fig. 1D) and the addition of Compound C at 1.0 × 10^−6^ M (Fig. 1E) significantly decreased the 2-DG uptake into C2C12 cells compared with HHW.

Western blot analysis showed that HHW significantly increased membrane Glut4 and phosphorylated AMPK (p-AMPK) in C2C12 cells (Fig. 1F, 1G). The bar graph represents the ratio of the each protein to actin protein bands quantified by densitometric analysis. There was no significant difference in total AMPK in C2C12 cells or membrane Glut2 in Hep G2 cells between treatments (Fig. 1H, 1I). These data indicate that the effect of H_2_ is in the muscle rather than in the liver.

### H_2_ Remains Higher in NHW and Saline Water than Pure Water

**Figure 2:**
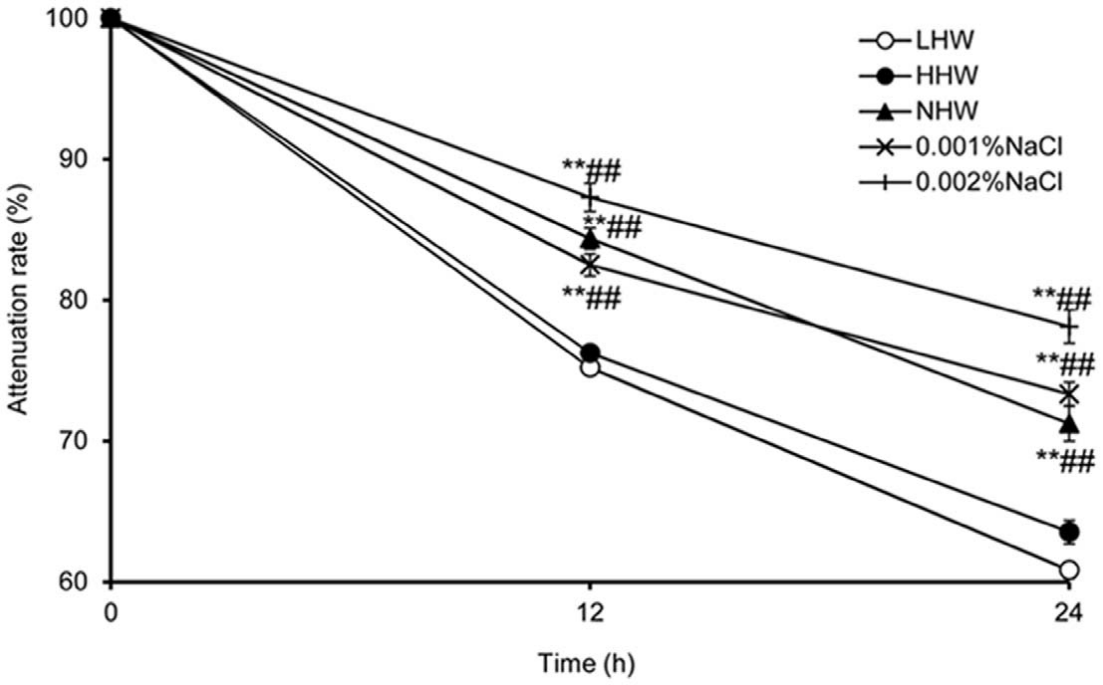
The H_2_ attenuation rate in pure water and several solutions. Natural hydrogen water (NHW) and 0.17 mM (0.001%) and 0.34 mM (0.002%) saline water with 0.8 mM H_2_ retained a higher hydrogen concentration than low-content (LHW) and high-content hydrogen water (HHW) at 12 and 24 hours (n = 7 for each group). Multiple comparisons were performed by Dunnett’s multipule comparison test. **P<0.01 vs LHW, ## *P*<0.01 vs HHW.

Figure 2 shows the H_2_ attenuation rate in LHW, HHW, NHW, and 0.17 mM (0.001%) and 0.34 mM (0.002%) saline water with 0.8 mM H_2_ for 24 hours. NHW, 0.17 mM saline water and 0.34 mM saline water had significantly higher H_2_ concentrations than LHW and HHW at 12 and 24 hours.

### The Antioxidant Effect of H_2_ is Weaker than N-acetyl-cysteine (NAC) and Vitamin C

The % antioxidant activity of H_2_ was significantly lower than NAC and vitamin C [H_2_ (n = 6), 47.22% ±5.60; NAC (n = 6), 80.25±2.62; vitamin C (n = 6), 91.62±1.42, respectively; *P*<0.01].

### Chronic i.p. Administration of H_2_ Improves Hyperglycemia in STZ-induced Diabetic Mice

**Figure 3:**
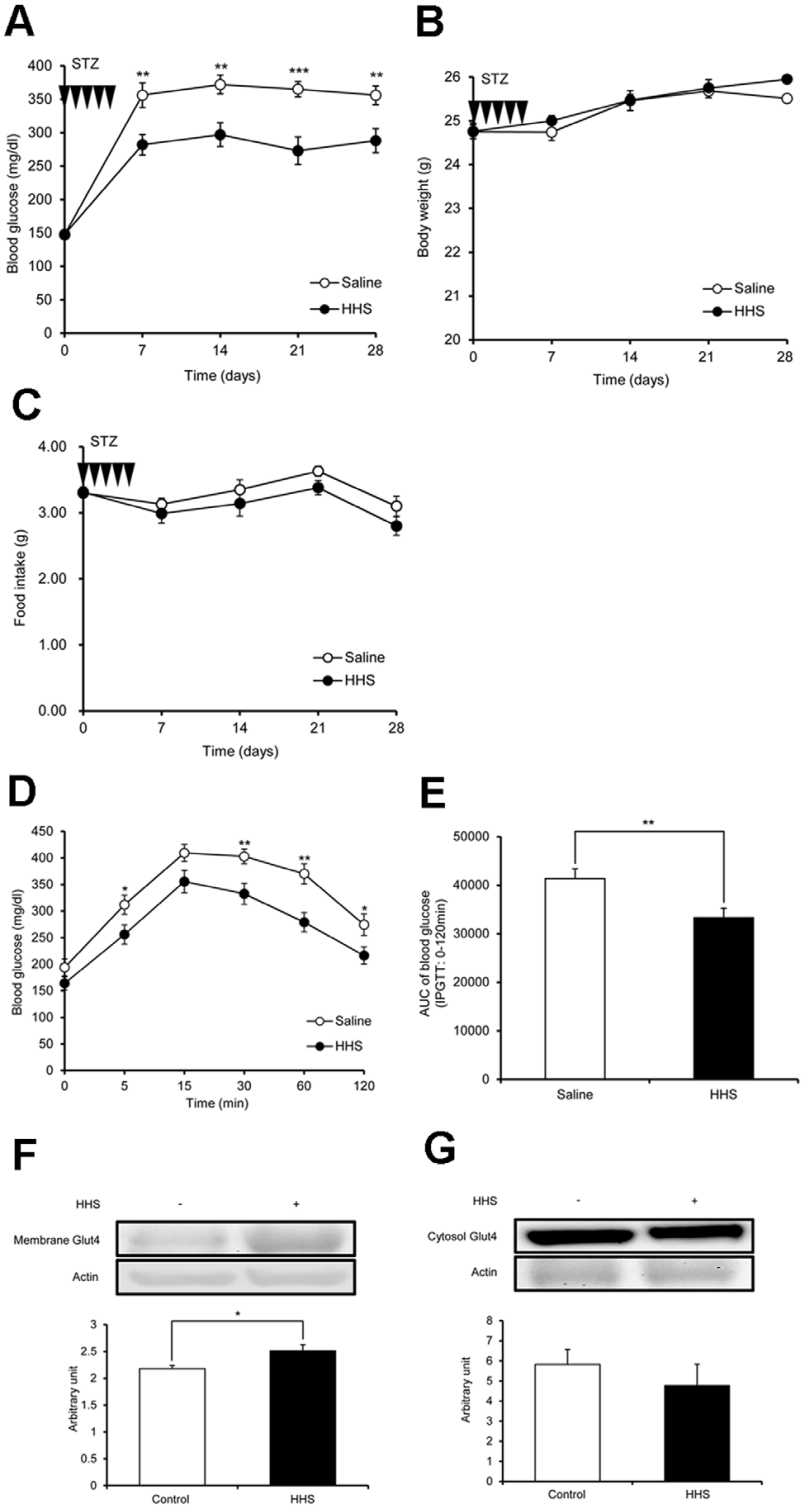
The effect of i.p. administration of H_2_ on hyperglycemia in STZ-treated mice. (A) Blood glucose in the group injected with high-content hydrogen saline (HHS) after STZ administration was significantly lower than the control group at every 7-days-interval measurement (n = 16 for each group). (B, C) Body weight and food intake every 7 day are shown. Although there was no significant difference in body weights or food intake between the control and HHS groups (n = 16 for each group), the food intake in the HHS group showed a tendency to decrease. (D, E) Blood glucose in the HHS group in the day-30 IPGTT was significantly lower than the control group at 5, 30, 60 and 120 min, and the area under the curve (AUC) of the HHS group was significantly lower than control (n = 16 for each group). (F) Membrane Glut4 in the HHS group was significantly increased compared to the control group, as determined by western blot analysis (n = 6 for each group). (G) Although there was no significant difference in cytosolic Glut4 between groups, the cytosolic Glut4 in the HHS group showed a tendency to decrease (n = 4 for each group). The bar graph shows the ratio of each protein to actin protein bands quantified by densitometric analysis. Comparisons with controls were performed by unpaired Student’s t test between two groups. *P<0.05, **P<0.01, ***P<0.001.

Blood glucose in the group injected with HHS after STZ administration was significantly lower than in the control (saline) group at every 7-day interval measurement (Fig. 3A). Body weights and food intake were monitored throughout the experimental period. There was no significant difference in body weight or food intake between the HHS and control groups (Fig. 3B, 3C). Blood glucose in the day-30 IPGTT was significantly lower in the HHS group than the control group at 5, 30, 60 and 120 min (Fig. 3D). In addition, the area under the curve (AUC) of the HHS group was significantly lower than the control group (Fig. 3E). In the western blot analysis of soleus muscle, membrane Glut4 was significantly upregulated in the HHS group compared to the control group (Fig. 3F), whereas cytosolic Glut4 showed a tendency to decrease in the HHS group, although this difference was not significant (Fig. 3G). Fasting plasma glucose, glycated albumin and triglyceride obtained on day 32 were significantly lower than the control group (Table 1). There was no significant difference in other parameters, including insulin and liver and kidney function tests.

**Table 1:** Laboratory investigations in the chronic i.p. administration of H_2_ experiment with STZ-induced T1DM mice.

	Saline	HHS
	n = 16	n = 16
Glucose (mg/dl)	316±19	255±19*
Glycated albumin (%)	8.13±0.45	5.80±0.66**
Insulin (ng/dL)	0.28±0.03	0.31±0.03
Total cholesterol (mg/dl)	93±3.0	92±2.2
HDL cholesterol (mg/dl)	46±1.6	48±1.0
Triglyceride (mg/dl)	32±3.7	23±1.7*
BUN (mg/dL)	21.53±2.46	17.56±0.52
Creatinin (mg/dL)	0.08±0.01	0.08±0.01
Total protain (g/dl)	4.67±0.06	4.81±0.06
Albumin (g/dl)	3.00±0.06	3.16±0.11
AST (IU/L)	119.13±8.30	137.19±14.6
ALT (IU/L)	28.13±1.58	25.31±1.48
γ-GTP (IU/L)	4.13±0.61	6.00±0.95

Data are expressed as mean ± standard error (SE). Statistical differences between groups were analyzed by Student’s *t*-test. STZ = streptozotocin; HHS = high content hydrogen saline; HDL = high-density lipoprotein; BUN = blood urea nitrogen; AST = aspartate aminotransferase; ALT = alanine aminotransferase; γ-GTP = γ-glutamyl transpeptidase.

* P<0.05,

** P<0.01.

### Chronic p.o. Administration of H_2_ Improves Diabetes in STZ-induced T1DM Mice

**Figure 4:**
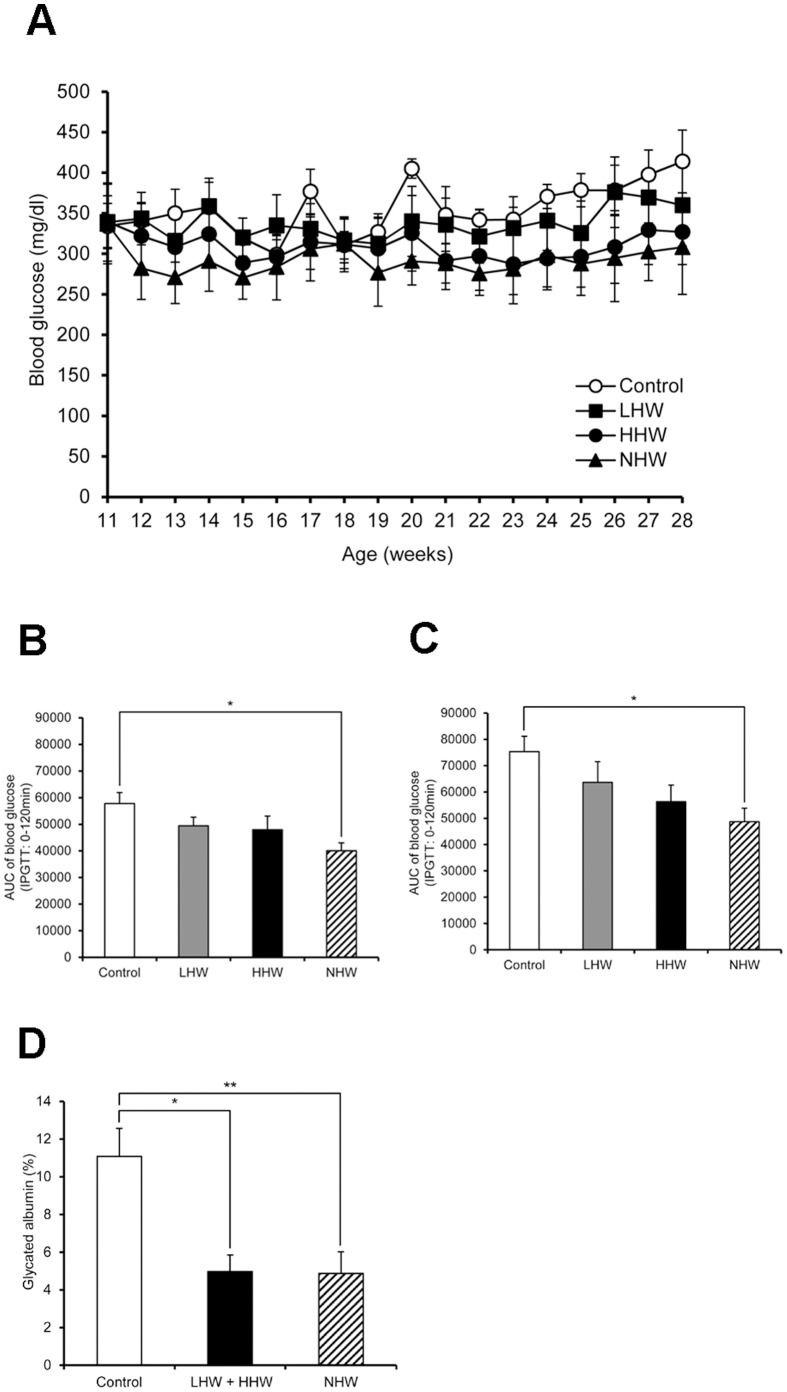
The effect of p.o. administration of H_2_ on diabetes in STZ-induced type 1 diabetic mice. (A) Blood glucose in the high content hydrogen water (HHW) and natural hydrogen water (NHW) groups at every weekly measurement showed a tendency to decrease compared to control, but, these decreases were not statistically significant (n = 6 for each group). (B, C) The AUC values of NHW group in the day-90 and day-120 IPGTTs were significantly decreased. Although the AUC values of the LHW, HHW groups in the day-90 and day-120 IPGTTs showed a tendency to decrease, these decrease were not statistically significant (n = 6 for each group). (D) As described in the Results, several mice were lost due to dehydration, so we combined the data of two groups [HHW (n = 2) and LHW (n = 2)] together. Glycated albumin in the combined LHW and HHW group (n = 4) and NHW group (n = 6) was significantly lower than the control group (n = 6). Comparisons with controls were performed by Dunnett’s multipule comparison test. *P<0.05, **P<0.01.

Blood glucose in the HHW and NHW groups showed a tendency to decrease, but these decreases were not statistically significant (Fig. 4A). The AUC values of the NHW group in the day-90 and day-120 IPGTTs were significantly decreased, although the decrease in the AUC values of the LHW and HHW groups did not reach statistical significance (Fig. 4B, C). On day 121, some mice in another animal room of our laboratory were found to be infected with mouse hepatitis virus (MHV). Although no mouse was infected with MHV in our animal room, we were unfortunately forbidden to change rooms and could not supply our mice with hydrogen water. Several mice were lost due to dehydration, and we therefore sacrificed all of the mice in this experiment at that time. We combined the data of two groups [LHW (n = 2) and HHW (n = 2)] together. The combined LHW and HHW group, as well as NHW group (n = 6), demonstrated markedly lowered glycated albumin levels compared with the control group (Fig. 4D). The triglyceride level in the combined LHW and HHW group and NHW group, as well as the non-esterified free fatty acid (NEFA) level in the NHW group significantly decreased compared with the control group (Table 2). There was no significant difference in any other parameter, such as insulin and liver and kidney function tests.

**Table 2:** Laboratory investigations in the chronic p.o. administration of H_2_ experiment with STZ-induced T1DM mice.

	Control	LHW+HHW	NHW
	n = 6	n = 4	n = 6
Glucose (mg/dl)	423±32	260±8	308±58
Glycated albumin (%)	11.10±1.83	4.98±0.88*	4.88±1.15**
Insulin (ng/dL)	0.24±0.05	0.43±0.07	0.42±0.11
Total cholesterol (mg/dl)	106±9.9	123±10.2	118±16.4
HDL cholesterol (mg/dl)	47±4.7	57±9.9	52±7.4
LDL cholesterol (mg/dl)	12±1.9	14±1.6	12±1.7
Triglyceride (mg/dl)	27±3.5	12±1.2**	16±2.0**
NEFA (µEq/L)	343±30	245±62	167±20**
BUN (mg/dL)	29.14±2.93	23.4±2.96	33.78±5.54
Creatinin (mg/dL)	0.12±0.01	0.12±0.01	0.12±0.02
Total protain (g/dl)	5.06±0.19	5.28±0.11	4.75±0.34
Albumin (g/dl)	2.94±0.19	3.28±0.75	2.95±0.35
AST (IU/L)	139±2.96	161±3.50	103±8.33
ALT (IU/L)	63±15.20	89±13.10	80±11.58
γ-GTP (IU/L)	4.20±0.74	4.50±0.89	5.00±1.23

Data are expressed as mean ± standard error (SE). Comparisons with control group were performed by Dunnett’s multiple comparison test. As described in Materials and Methods, we lost several mice accidentally and therefore combined two groups [LHW group (n = 2) and HHW group (n = 2)] data together. STZ = streptozotocin; T1DM = type 1 diabetes mellitus; LHW = low content hydrogen water; HHW = high content hydrogen water; NHW = natural hydrogen water; HDL = high-density lipoprotein; LDL = low-density lipoprotein; NEFA = free fatty acids; BUN = blood urea nitrogen; AST = aspartate aminotransferase; ALT = alanine aminotransferase; γ-GTP = γ-glutamyl transpeptidase.

* P<0.05,

** P<0.01.

**Figure 5:**
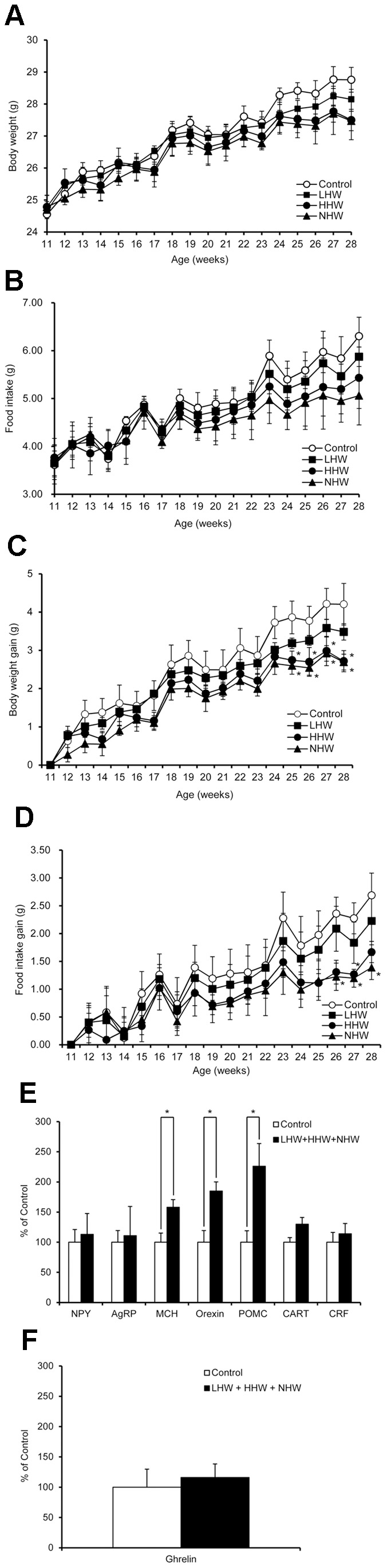
The effect of p.o. administration of H_2_ on food intake in STZ-induced type 1 diabetic mice. (A, B) Body weights and food intake were measured every 7 days. Although there was no significant difference in body weight or food intake among the 4 groups (n = 6 for each group), the food intake in the LHW, HHW and NHW groups showed a tendency to decrease in the latter part of this experiment. (C) The body weight gain in the HHW group (n = 6) and NHW group (n = 6) was significantly lower than the control group (n = 6) at 25, 26, 27, and 28 weeks of age. (D) The food intake gain in the HHW group (n = 6) was significantly lower than the control group (n = 6) at 27 weeks of age. Additionally, the food intake gain in the NHW group (n = 6) was significantly lower than the control group (n = 6) at 26, 27, and 28 weeks of age. (E) Orexigenic melanin-concentrating hormone (MCH) and orexin mRNA and anorexigenic pro-opiomelanocortin (POMC) mRNA expressions in the hypothalamus were significantly increased in the combined LHW, HHW, and NHW group (n = 10) compared with the control group (n = 6). (F) There was no significant difference in ghrelin mRNA in the stomach (the control group, n = 6; the combined LHW, HHW, and NHW group, n = 10). Comparisons with controls were performed by Dunnett’s multipule comparison test. *P<0.05.

The food intake and body weight in the LHW, HHW and NHW groups showed a tendency to decrease in the course of the experiment (Fig. 5A, 5B). HHW and NHW significantly decreased body weight gain at 25, 26, 27, and 28 weeks of age (Fig. 5C). HHW significantly decreased food intake gain at 27 weeks of age and NHW significantly decreased food intake gain at 26, 27, and 28 weeks of age (Fig. 5D). Therefore, we examined the potential changes of the expression of the hypothalamic feeding-regulatory peptides. Orexigenic MCH and orexin mRNA and anorexigenic POMC mRNA expressions in the hypothalamus during fasting were significantly increased in the combined LHW, HHW, and NHW group compared with the control group. (Fig. 5E). There was no significant difference in other hypothalamic peptide as well as gastric ghrelin mRNA expression (Fig. 5F).

### Chronic p.o. Administration of H_2_ has no Effect on Diabetes in High-fat Diet-induced T2DM Mice

The HHW group had a significantly decreased AUC in the day-90 IPGTT. However, there were no significant differences in blood glucose in the weekly measurements, in the AUC values of the day-30, -60, -120, -150, or -180 IPGTT, or in glycated albumin levels between the control and HHW groups (Table 3). Each group showed similar hyperinsulinemia.

**Table 3:** Laboratory investigations in the chronic p.o. administration of H_2_ experiment with high-fat diet-induced T2DM mice.

		Control	HHW	NHW
		n = 7	n = 7	n = 7
Blood glucose (mg/dl)				
	Age (weeks)			
	9	167±7	173±10	174±8
	11	185±5	207±9	181±8
	13	165±8	154±5	153±8
	15	181±9	179±8	165±7
	17	180±8	177±5	170±7
	19	176±6	167±7	173±7
	21	198±11	174±6	193±9
	23	217±4	215±8	215±8
	25	213±7	219±6	203±8
	27	204±9	199±2	207±6
	29	224±10	229±5	231±8
	31	220±9	229±7	231±7
AUC (IPGTT)				
	Time (days)			
	30	30599±1266	30659±1173	29823±1318
	60	33436±1423	32931±1429	30519±1299
	90	35506±2370	26080±1984**	32481±2830
	120	36876±3910	35468±2749	36813±2459
	150	37112±3308	36244±1918	34441±1526
	180	35769±2773	35258±1398	34223±1134
Laboratory investigations				
	Blood glucose (mg/dl)	233±10	247±10	244±6
	Glycated albumin (%)	2.84±0.15	2.70±0.30	2.70±0.35
	Insulin (ng/ml)	4.09±0.52	5.46±0.78	5.19±0.72
	Total cholesterol (mg/dl)	185±11.7	197±8.0	175±11.7
	HDL cholesterol (mg/dl)	75±2.9	83±2.7	74±2.8
	LDL cholesterol (mg/dl)	11±1.4	11±1.1	10±2.3
	Triglyceride (mg/dl)	25±2.6	20±1.9	30±5.0
	NEFA (µEq/L)	343±14	346±25	357±23

Data are expressed as mean ± standard error (SE). Comparisons with control group were performed by Dunnett’s multipule comparison test. T2DM = type 2 diabetes mellitus; HHW = high content hydrogen water; NHW = natural hydrogen water; AUC = area under the curve; IPGTT = intraperitoneal glucose tolerance test; HDL = high-density lipoprotein; LDL = low-density lipoprotein; NEFA = free fatty acids.

** P<0.01.

### Chronic p.o. Administration of H_2_ has no Effect on Diabetes in Type 2 Diabetic *db/db* Mice

There were no significant differences in blood glucose at the weekly measurements, in the AUC values of the day-30, -60, -90, or -120 IPGTT, or in glycated albumin levels between the control and HHW groups (Table 4). Several mice were lost by natural death from unknown causes after 120 days, so we sacrificed all of the mice in this experiment at that time. Each group showed similar hyperinsulinemia.

**Table 4:** Laboratory investigations in the chronic p.o. administration of H_2_ experiment with type 2 diabetic *db/db* mice.

		Control	HHW	NHW
		n = 7	n = 7	n = 7
Blood glucose (mg/dl)				
	Age (weeks)			
	9	293±12	298±23	296±14
	11	340±10	314±14	328±10
	13	347±9	324±12	359±17
	15	359±17	333±33	363±10
	17	353±18	319±16	353±11
	19	313±26	376±13	414±19
	21	398±17	348±16	403±32
	23	404±31	357±37	409±26
	25	371±14	349±22	403±18
AUC (IPGTT)				
	Time (days)			
	30	60718±3687	60420±2202	56646±2795
	60	60503±2019	63120±3343	61514±1535
	90	64998±2338	59567±2842	62208±2082
	120	62841±2173	59295±2052	67816±3753
Laboratory investigations				
	Blood glucose (mg/dl)	439±17	400±65	479±11
	Glycated albumin (%)	6.38±0.64	5.40±1.00	6.95±0.49
	Insulin (ng/ml)	3.49±0.69	3.61±0.49	2.89±0.25
	Total cholesterol (mg/dl)	91±15.0	100±6.2	106±6.2
	HDL cholesterol (mg/dl)	58±8.8	62±2.8	64±4.5
	LDL cholesterol (mg/dl)	5±0.7	6±1.6	8±1.7
	Triglyceride (mg/dl)	24±2.3	29±8.8	34±3.5
	NEFA (µEq/L)	1096±56	1121±137	1331±123

Data are expressed as mean ± standard error (SE). Comparisons with control group were performed by Dunnett’s multiple comparison test. As described in Materials and Methods, we lost several mice by natural death after 120 days in each group and thus the number of mice for laboratory investigations are 6 (control), 4 (HHW) and 6 (NHW). HHW = high content hydrogen water; NHW = natural hydrogen water; AUC = area under the curve; IPGTT = intraperitoneal glucose tolerance test; HDL = high-density lipoprotein; LDL = low-density lipoprotein; NEFA = free fatty acids.

### STZ-induced T1DM Mice Show the Highest Water Intake

The control water intake of STZ-induced T1DM mice and high-fat diet-induced T2DM and *db/db* mice were 19.94±0.75 (n = 6), 4.24±0.20 (n = 7), 15.01±0.67 (n = 7) ml/day, respectively. The NHW group showed no difference to the controls: 19.21±1.82 (n = 6, STZ), 3.92±0.18 (n = 7, high-fat), and 15.42±0.74 (n = 7, *db/db*) ml/day of water intake. The high-fat diet-induced T2DM mice did not differ significantly compared to normal mice in water intake (data not shown). Although the HHW group appeared not to be different from the NHW, the existence of dropped water due to the H_2_ bubbles made it difficult to measure correctly the water intake of this animal group.

## Discussion

The gold-standard treatment for patients with T1DM remains intensive insulin therapy that should be provided in multiple daily injections or by continuous subcutaneous insulin infusion, complemented with frequent blood glucose monitoring. Advances in the understanding of the insulin molecule as well as the development of new devices for insulin administration have allowed treatment regimens to more closely mimic the physiologic insulin response of healthy individuals. However, various shortcomings of insulin therapies, such as the injectable nature of insulin, the development of insulin resistance, and the inability to reach excellent glycemic control due to hyperglycemia and hypoglycemia, have motivated researchers to develop noninsulin pharmacological therapies to manage T1DM. These include, but not are limited to, immunotherapeutic agents, incretin-based therapies, recombinant human insulin-like growth factors, stem cells, and the transplantation of pancreatic islets. Although some therapies, either independently or as adjuvant to insulin, are currently used to manage T1DM, most therapies are in developmental stages and/or are limited to use. Therefore, therapeutic alternatives to insulin that are freely available are needed [23], [24].

Our study demonstrates for the first time that H_2_ was effective in improving glycemic control in a STZ-induced type 1 diabetic animal model without producing hypoglycemia. This was achieved not only by intra-peritoneal administration of H_2_ but also by oral administration, despite the unstable nature of H_2_ in the water. Addition of salts such as sodium chloride could help maintain H_2_ concentrations in the water, as in NHW. Neither gross behavioral abnormalities nor apparent biochemical changes such as liver and kidney functions were observed during the 1–4-month experimental period. These results indicate that H_2_ therapy could overcome several drawbacks associated with insulin therapy.

H_2_ is a potent scavenger of reactive oxygen species (ROS). Both increased generation of ROS and impaired antioxidant defenses cause oxidative stress which is the process of cellular injury. The mitochondrial electron transport chain is the main source of ROS in most cells [25]. Donating one electron to molecular oxygen results in the formation of superoxide (O_2_^−^) [26]. In physiological homeostasis, O_2_^−^ is converted to hydrogen peroxide (H_2_O_2_) by the enzyme superoxide dismutase (SOD) and H_2_O_2_ is converted into water by the enzymes catalase or glutathione peroxidase [27]. In the presence of reduced transition metals (e.g., ferrous or cuprous ions), H_2_O_2_ can be converted into the most highly reactive hydroxyl radical (·OH) [28].

Not only H_2_, but also N-acetyl-cysteine (NAC) and vitamin C are well known as potent scavengers. As mentioned above, H_2_ selectively reduces the highly toxic ·OH in vitro [10]. The selective deoxidization of H_2_ is due to its mild reducing power [10]. We verified that H_2_ is a weak scavenger more than NAC and vitamin C in this study. Recently, several studies suggest that H_2_ increases the amount of SOD, catalase and heme oxygenase-1 [17], [29], [30]. NAC directly scavenges H_2_O_2_ and ·OH in vitro [31]. NAC also decreases free radical levels by increasing the glutathione synthesis [32], [33]. NAC has historically been used as a mucolytic agent in a variety of respiratory illnesses. NAC also have beneficial effects in conditions characterized by decreased glutathione or oxidative stress, such as HIV infection, cancer, heart disease, and cigarette smoking [34]. However, there are references in the literature implicating NAC in the oxidative damage of biological systems both in vitro [35]–[37] and in vivo [38], [39], depending on the experimental conditions, such as the presence or absence of transition metal ions. Vitamin C directly scavenges O_2_^−^, H_2_O_2_ and ·OH in vitro [10]. Although vitamin C has been credited with benefits in many human diseases such as atherosclerosis and cancer [40], [41], vitamin C may act as a pro-oxidant due to the high reactivity of vitamin C with transition metals, including iron [42], [43]. In addition, a human study suggests that supplementation with both NAC and vitamin C increases oxidative stress and tissue damage [39]. Few reports have described unwanted side effects of H_2_. The reason may be due to its mild antioxidant effect and H_2_ appears not to disturb physiological metabolic oxidation-reduction reactions or disrupt ROS involved in cell signaling [10].

SOD has a pivotal role in the detoxification of ROS [44]. In C2C12 cells, it has been documented that the exposure to 2 mM of H_2_O_2_ did not reduce SOD activation [45]. On the other hand, the exposure to 1 mM of H_2_O_2_ diminished SOD activation by almost 50% in HepG2 cells [46]. These reports indicate that the antioxidant capacity of SOD in C2C12 cells is larger than HepG2 cells. Thus, REDOX state in cell seems lower in liver than that in muscle. Our results in C2C12 cells and HepG2 cells experiments indicate that the effect of H_2_ in Glut4 translocation may be independent of the antioxidant effect.

Up to 75% of insulin-dependent glucose disposal occurs in skeletal muscle [47]. Insulin stimulates glucose uptake in skeletal muscle by promoting translocation of Glut4 from intracellular sites to the plasma membrane. Mouse C2C12 cells, derived from the mouse skeletal muscle C2 cell line, possess morphological, biochemical and metabolic properties similar to isolated skeletal muscle [48]. In this study, H_2_ promoted 2-DG uptake into C2C12 cells by stimulating Glut4 translocation. This *in vitro* experiment also indicated that the Glut4 translocation was stimulated by at least PI3K, PKC, and AMPK signaling. PKC is classified into three groups according to activation node: conventional isoforms (α, β1, β2, and γ), novel isoforms (δ, ε, θ, and η), and atypical isoforms (ζ, λ/τ). Unlike conventional or novel PKCs, atypical PKC isoforms (aPKCs) have been suggested to act as downstream mediators of PI3K and play roles in insulin-stimulated glucose uptake and Glut4 translocation in adipocytes and skeletal muscle [49]. Therefore, the increased expression of PKC isoforms in this study might have been aPKCs, although we did not directly measure aPKCs.

**Figure 6:**
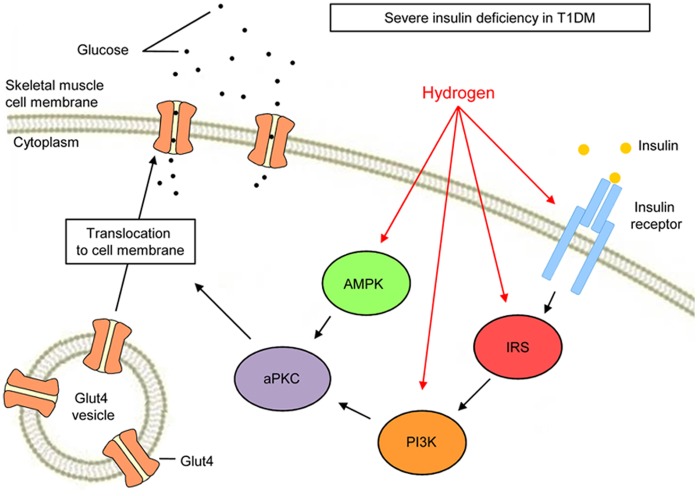
The hypothetical model of H_2_ action in glucose excursion. H_2_ promotes glucose uptake into skeletal muscle by stimulating Glut4 translocation by activating phosphatidylinositol-3-OH kinase (PI3K), atypical protein kinase C (aPKC), and AMP-activated protein kinase (AMPK) under conditions of severe insulin deficiency. H_2_ has little effect on glucose excursion under conditions of hyperinsulinemia and insulin resistance.

AMPK plays critical roles in regulating growth and reprogramming metabolism and has recently been implicated in autophagy and cell polarity [50]. An important physiological process that AMPK promotes in skeletal muscle is glucose transport. This glucose transport occurs in the absence of insulin and also under the regulation of the insulin-responsive Glut4 [51]. In muscle preparations of rodents and cultured L6 myotubes, two substances that activate AMPK, 5-aminoimidazole-4-carboxamide-1-β-D-riboside (AICAR) (which enters the cell and mimics 5′-AMP) and dinitrophenol (DNP) (which uncouples mitochondrial oxidative phosphorylation, thereby increasing 5′-AMP levels), also activate aPKCs. aPKCs are required for increases in Glut4 translocation to the plasma membrane and glucose transport during AICAR and DNP stimulation in L6 myotubes [52]. This activation of aPKCs by AMPK occurs in the absence of PI3K signaling. Therefore, H_2_ may stimulate Glut4 translocation via the activation of aPKC, either by stimulating PI3K, activating signaling upstream of PI3K or activating AMPK signaling (Fig. 6). The signaling events may occur in a linear or parallel fashion with the latter converging upon Glut4 translocation.

STZ is synthesized by *Streptomyces achromogenes* and is widely used to induce experimental diabetes in animals. STZ is taken up by pancreatic β-cells via Glut2. Although STZ is a nitric oxide (NO) donor and NO contributes to DNA damage in β-cells [53], [54], recent reports have shown that the main reason STZ induces β-cell death is alkylation of DNA [55], [56]. NO possesses comparatively weak reactivity among ROS. Therefore, H_2_, which selectively reduces the strongest ROS, is unlikely to prevent the STZ-induced β-cell death as a therapeutic antioxidant. Indeed, the fasting plasma insulin levels in this chronic i.p. experiment with STZ-induced diabetic mice showed no significant difference between the control and HHS groups. Specifically, the results in the chronic i.p. and p.o. administration of H_2_ experiments in STZ mice suggest that the improvement of hyperglycemia and is due to the promotion of glucose uptake into skeletal muscle by stimulating Glut4 translocation.

Hyperphagia is a classical manifestation of uncontrolled diabetes, but the pathogenesis remains incompletely understood. Insulin receptor (IR) is expressed in most tissues of the body, including the neuronal tissue of the central nervous system (CNS). In the CNS, the IR displays distinct patterns of expression in the olfactory bulb, the hypothalamus, and the pituitary [57], [58]. Insulin dysfunction in CNS could be a candidate for causes of diabetic hyperphagia, because mice in a neuron-specific knockout of the IR show increased food intake and body weight [59]. Uncontrolled insulin-deficient diabetes induced by STZ is an established model of sustained hyperphagia in rodents. In our model of STZ-induced T1DM mice with chronic p.o. H_2_ administration, the food intake and body weight gain are significantly blunted in the treatment group. Therefore, H_2_ may have an inhibitory effect on feeding of T1DM mice as with insulin.

Neuropeptides in the hypothalamus play a pivotal role in physiologic mechanisms regulating food intake and body weight [60]–[62]. In the arcuate nucleus (ARC) in the hypothalamus, which specially plays a key role in regulating food intake, there are two neuronal populations with opposing effects on food intake: neurons which co-express NPY and AgRP stimulate food intake, whereas POMC neurons suppress feeding. A major mechanism underlying the effects of insulin to reduce food intake involves the regulation of hypothalamic neuropeptide systems [62]. Specifically, insulin inhibits NPY/AgRP neurons and activates POMC neurons [62]. Ghrelin is an important brain–gut peptide, an endogenous ligand of the growth hormone secretagogue (GHS) receptor from the stomach, and the first orexigenic peptide of the periphery [61], [63]. Ghrelin increases food intake and body weight by activating hypothalamic NPY/AgRP neurons [64]. A lack of meal-induced ghrelin suppression caused by severe insulin deficiency is considered one of the reasons for hyperphagia in uncontrolled T1DM [65]–[67]. In our chronic p.o. administration of H_2_ experiment with STZ-induced T1DM mice, anorexigenic POMC mRNA and orexigenic MCH and orexin mRNA expressions in the hypothalamus were significantly increased in the H_2_ treatment group compared with the control group. On the other hand, there was no significant difference in orexigenic ghrelin mRNA expression in the stomach. These results suggest that H_2_-induced anorexigenic effects may be mediated by POMC, and MCH and orexin be involved in other motivational behaviors that need to be clarified. It is to be determined whether or not POMC is involved in improvement on hyperglycemia in our model since POMC regulates glucose homeostasis and insulin sensitivity through distinct CNS populations from those regulating food intake and body weight [68].

In this study, we used two types of obese and T2DM mouse models: high-fat diet-induced diabetic mice and *db/db* mice. The H_2_ treatment had little effect on these animal models with hyperinsulinemia and insulin resistance: neither fasting glucose nor glycated albumin differed significantly in high-fat diet-induced diabetic or *db/db* mice. Although the origin of insulin resistance has been difficult to elucidate in part due to the diverse set of risk factors linked to this condition, the impaired insulin-stimulated glucose transport in muscle and adipose tissue is considered a major contributor to the pathogenesis of insulin-resistant states such as obesity and T2DM [69]. The marked contrast in the effect of H_2_ in our T1DM and T2DM animal models suggest that H_2_ may converge on the insulin signals in the cells and not improve insulin sensitivity. The hyperglycemia causes a rise in serum osmolarity and thirst with polydipsia. STZ-induced diabetic animal models show very severe hyperglycemia and suffer from chronic polydipsia more than the T2DM mouse models, which may account in part for the difference in the effectiveness of H_2_ to reduce hyperglycemia.

In contrast to our data, Kamimura et al reported that 3-months p.o. administration of H_2_ water (0.8 mM) to *db/db* mice decreased plasma glucose, insulin and triglyceride levels, stimulated energy metabolism, and, as a result, suppressed the gain of fat and body weight [16]. H_2_ attenuation was considered one of the reasons for these differences. The glass vessel used by Kamimura retained almost 100% of the original H_2_ in HHW after 24 hours, whereas our glass vessel retained only 64% of the H_2_ in HHW or 71% H_2_ in NHW after 24 hours. Kajiyama et al reported that 8 weeks p.o. administration of hydrogen-rich water (0.6 mM) did not improve plasma glucose, insulin, HbA_1c_ or body weight in patients with T2DM or impaired glucose tolerance [17]. Our data are in line with their clinical data. The difference in H_2_ administration methods may also have influenced the results, although our study indicates both i.p. and p.o. administration routes are effective in a T1DM model. Further studies are required to evaluate the molecular mechanisms of H_2_ and the effect of H_2_ treatment on T1DM and T2DM patients.

Much of the mortality of type 1 and type 2 diabetes results from long-term complications of microvascular (nephropathy, retinopathy, and neuropathy) and macrovascular (ischemic heart disease, peripheral vascular disease, and stroke) events. However, the onset and progression of diabetes complications correlates substantially with glycemic control. Therefore, the present findings of improved glycemic control by oral administration of H_2_ strongly suggest that H_2_ is a novel therapeutic molecule that could aid in type 1 diabetes management. Because the occurrence and progression of diabetes complications is influenced by the presence and degree of hypertension and dyslipidemia [70], the potential beneficial effects of H_2_ treatment on these nonglycemic risk factors [16], [17], including the improvement of triglyceride and free fatty acid levels in our study, are important and remain to be clarified in T1DM patients.

Previous study demonstrated that H_2_ could be detected in the venous blood of rats at the level of 5 µM after 3 min of administration of saturated (0.8 mM) H_2_ water into the stomach [71]. The concentration of H_2_ used in the study was similar to that in our study. H_2_ almost does not exist in artery but exist in vein around the level of 1 µM in the physiological condition of rats [10], [72]. Even the low concentration (0.04 mM) of H_2_ in drinking water was effective in the treatment of mouse model of Parkinson’s disease [13]. The increased water intake together with these previous data indicates that ingested H_2_ could move to circulation and reach the target sites in our diabetic animal models.

In conclusion, our study demonstrates that H_2_ stimulates Glut4 translocation and glucose uptake into skeletal muscle and may be a novel therapeutic alternative to insulin in T1DM that can be administered orally.
