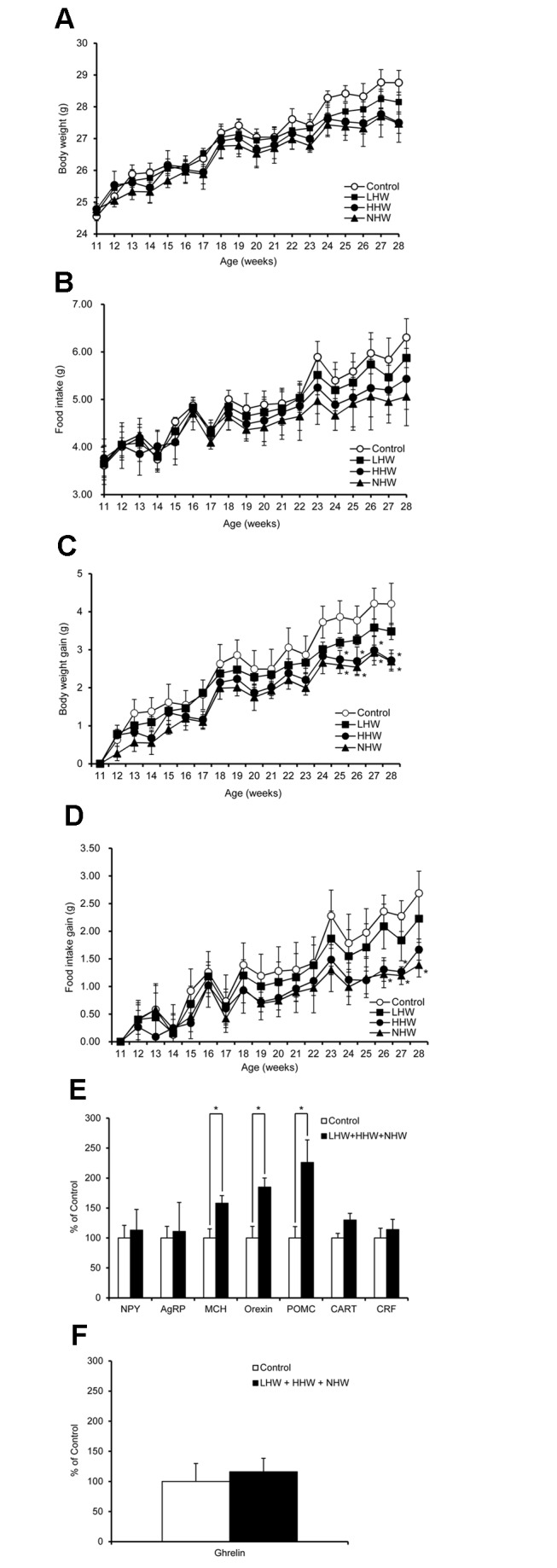# Correction: Hydrogen Improves Glycemic Control in Type1 Diabetic Animal Model by Promoting Glucose Uptake into Skeletal Muscle

**DOI:** 10.1371/annotation/ea26285b-dda3-470d-a8df-241df9fbc5ad

**Published:** 2013-04-22

**Authors:** Haruka Amitani, Akihiro Asakawa, Kaichun Cheng, Marie Amitani, Kaori Kaimoto, Masako Nakano, Miharu Ushikai, Yingxiao Li, Minglun Tsai, Jiang-Bo Li, Mutsumi Terashi, Huhe Chaolu, Ryozo Kamimura, Akio Inui

Figure 5 was omitted from the PDF. The figure is available here: 

**Figure:**